# Preserved in vitro immunoreactivity in children receiving long-term immunosuppressive therapy due to inflammatory bowel disease or autoimmune hepatitis

**DOI:** 10.1186/s40348-018-0079-0

**Published:** 2018-01-19

**Authors:** Teresa Schleker, Eva-Maria Jacobsen, Benjamin Mayer, Gudrun Strauss, Klaus-Michael Debatin, Carsten Posovszky

**Affiliations:** 1 Department of Pediatrics and Adolescent Medicine, Ulm University Medical Center, Ulm, Germany; 2 Institute of Epidemiology and Medical Biometry, Ulm University, Ulm, Germany; 3 Pediatric Gastroenterology and Nutrition, Department of Pediatrics and Adolescent Medicine, Ulm University Medical Center, Eythstr. 24, 89075 Ulm, Germany

**Keywords:** Vaccination, Live vaccine, Immunosuppression, Inflammatory bowel disease, Autoimmune hepatitis, Lymphocyte proliferation

## Abstract

**Background:**

Children with inflammatory bowel disease (IBD) or autoimmune hepatitis (AIH) are at risk for severe infections. This is partially a result of their chronic disease condition but, moreover, a side effect of their immunosuppressive therapy. Currently, vaccinations with live vaccines are regarded as contraindicated under immunosuppressive therapy, mainly because of concerns about side effects and a lack of data showing an adequate immune reaction. As there is no systematic study on the individual immunoreactivity under immunosuppressive therapy in this patient group, we analyzed the lymphocyte subgroups and immunoreactivity of lymphocytes in children with IBD or AIH with and without immunosuppressive therapy in vitro.

**Methods:**

We collected whole blood samples from 17 children with IBD or AIH on high-level immunosuppression (IS) (group 1) and 8 on low-level IS (group 2) in comparison with 6 patients without systemic IS (group 3). After Ficoll separation of peripheral mononuclear cells, the samples were analyzed by flow cytometry to determine the lymphocyte subgroups. Furthermore, we stimulated the isolated lymphocytes with phytohemagglutinin (PHA), tetanus antigen, and adenovirus antigen and measured their proliferation by incorporation of H_3_-thymidine detected in a beta counter.

The statistical evaluation was performed by Kruskal-Wallis test and Mann-Whitney *U* test using a bilateral level of significance of *α* = 5%.

**Results:**

Patients with low- or high-level IS showed no significant difference in the number of lymphocytes or T cells.

Interestingly, IS did not influence the lymphocyte proliferation assay significantly regarding median reaction to PHA, tetanus antigen, or adenovirus antigen between the three groups. However, comparing all immunosuppressed patients to the patients without IS, there was a significant difference towards stimulation with tetanus antigen.

**Conclusions:**

Contrary to expectations of a strong influence of IS therapy on the immunoreactivity, this study showed only minor differences between the groups with high-level, low-level, and no IS. Particularly, the in vitro reactivity to adenovirus antigen was nearly the same in all three groups. We assume that—provided a normal distribution and count of lymphocyte subgroups—patients with moderate immunosuppression might be capable of raising an effective immune response to inactivated and live vaccines.

**Electronic supplementary material:**

The online version of this article (10.1186/s40348-018-0079-0) contains supplementary material, which is available to authorized users.

## Background

There are different reasons why children and adolescents with inflammatory bowel disease (IBD) and autoimmune hepatitis (AIH) underlie a high risk for severe infections. First, those diseases are immune-mediated; thus, the disease itself is responsible for changes in immune reactions. Secondly, the treatment of immune-mediated diseases requires pharmacological immunosuppression (IS) [1–3]. Moreover, the rates of vaccination are not always high enough to ensure herd immunity for immunosuppressed children [4]. Thus, young patients with IBD and AIH lacking vaccinations are at higher risk for severe infections with for example varicella [5].

Vaccinations with live vaccines, as varicella and measles vaccines, are currently not recommended in patients receiving IS therapy. The main reasons are safety concerns due to a lack of data showing a sufficient reaction to the vaccination without higher complication rates. Thus, vaccinations are suggested to be administered before starting IS therapy or therapy should be interrupted during remission of the disease [6]. However, already at diagnosis, IS therapy needs to be started and IS interruption is often impossible for satisfying disease control.

The ability of the adaptive immune system to react adequately on viral infections or vaccinations depends on a functional B and T lymphocyte compartment.

Vaccine-induced effectors are antibodies produced by B lymphocytes capable of binding toxin or antigen and cytotoxic CD8+ T lymphocytes (CTL) that recognize and kill infected cells [7]. Thus, activation of T and B lymphocytes by activated dendritic cells and monocytes is the initial step of a vaccine response. Besides the predominant role of B cells during this process, the importance of T cell response is evident: T cells are essential for the induction of high-affinity antibodies and immune memory. Determination of the altered immune competence in patients receiving immunosuppressive drugs depends on the general condition and the degree and mode of action of the immunosuppressive medication [8].

A proliferation assay is a simple in vitro method of measuring lymphocyte activation and proliferation ability. Lymphocytes can be artificially stimulated using unspecific mitogens, e.g., phytohemagglutinin (PHA) or antigens that induce specific recall T cell responses either through natural exposure (adenovirus antigen) or as a result of vaccination (tetanus antigen).

Therefore, it is important to examine the individual immunoreactivity in children and adolescents with IBD and AIH to provide a deeper insight in the functional capability of the cellular immune system under different regimes of IS therapy. This may influence the decision on vaccinations during IS therapy with live vaccines in these patients.

## Methods

We collected blood samples of 31 patients aged 3 to 18 years treated in the pediatric gastroenterology of Ulm University Medical Center as part of the VARICED study investigating varicella vaccination in immunosuppressed patients with IBD (according to the ethics approval by the ethics committee, University of Ulm, number 214/2013). Sixteen of them are female, and 15 are male. Sixteen patients have Crohn’s disease (CD), 12 ulcerative colitis (UC), and 3 AIH (see Table 1).

**Table 1:** Patient characteristics

Group	Gender	Age	Diagnosis	Age at diagnosis	IS
1	f	9	CD	3	Sirolimus 0.1 mg/kg/day (10.4 ng/ml blood level)
1	m	14	UC	11	Vedolizumab 300 mg/8 weeks
1	m	17	UC	15	Adalimumab 40 mg/week (11.57 μg/ml blood level)
1	f	12	CD	8	Adalimumab 40 mg/week (10.35 μg/ml blood level)
1	m	16	CD	3	Adalimumab 40 mg/2 weeks (6.5 μg/ml blood level)
1	m	14	UC	12	Adalimumab 40 mg/week (12.35 μg/ml blood level) Tacrolimus 0.12 mg/kg/day (2.6 ng/ml blood level) AZA 1.0 mg/kg/day
1	f	11	UC	10	Infliximab 5 mg/kg/4 weeks (4.66 μg/ml blood level)
1	f	18	UC	17	Vedolizumab 300 mg/8 weeks AZA 2.6 mg/kg/day Sirolimus 0.18 mg/kg/day (8.8 ng/ml blood level) Prednisolone 0.5 mg/kg/day
1	f	15	UC	10	Adalimumab 40 mg/4 weeks (6.5 μg/ml blood level)
1	m	5	CD	3	Infliximab 10 mg/kg/4 weeks (0.5 μg/ml blood level) Tacrolimus 0.19 mg/kg/day (30.9 ng/ml blood level)
1	f	14	CD	13	Infliximab 5 mg/kg/4 weeks (3.36 μg/ml blood level)
1	m	14	CD	13	Infliximab 5 mg/kg/6 weeks (1.56 μg/ml blood level) AZA 2.1 mg/kg/d
1	m	9	UC	6	Infliximab 5 mg/kg/6–8 weeks (1.03 μg/ml blood level) AZA 2.2 mg/kg/day
1	f	10	UC	5	Vedolizumab 250 mg/8 weeks
1	m	12	CD	12	Infliximab 5 mg/kg/8 weeks (0.94 μg/ml blood level)
1	m	11	CD	4	Golimumab 50 mg/4 weeks (8.5 μg/ml blood level)
1	m	15	CD	13	Infliximab 5 mg/kg/4 weeks (14.2 μg/ml blood level)
2	f	10	CD	8	AZA 2.3 mg/kg/day
2	f	9	UC	1	AZA 2.2 mg/kg/day
2	m	18	CD	16	AZA 1.9 mg/kg/day
2	f	17	CD	3	AZA 1.9 mg/kg/day
2	f	13	AIH	4	AZA 1 mg/kg/day
2	m	17	AIH	10	AZA 1.7 mg/kg/day
2	f	16	AIH	12	AZA 1.1 mg/kg/day
2	f	9	UC	5	Tacrolimus 0.23 mg/kg/day (4.5 ng/ml blood level)
3	f	13	CD	12	No IS
3	m	15	CD	3	No IS
3	f	3	CD	3	No IS
3	m	14	UC	2	No IS
3	f	15	UC	10	No IS
3	m	12	CD	12	No IS

Blood samples were taken in additional blood collection tubes during regularly needed blood examinations.

Peripheral blood mononuclear cells (PBMC) were separated using a Ficoll gradient separation (Biocoll Separating Solution, Biochrom-AG, Germany). After counting, PBMCs were stored in liquid nitrogen.

PBMCs (10^6^ cells/ml; 100 μl per well) were stimulated with PHA (Invitrogen, Germany; final concentration 15 μl/ml), tetanus antigen (Pharmore, Germany; final concentration 50 μl/ml), and adenovirus antigen (Institute of Virology, Ulm University Medical Center, Germany; final concentration 20 μl/ml). For PHA and tetanus antigen, we used culture medium as negative control, and for adenovirus antigen, we used HFF culture supernatant as negative control (Institute of Virology, Ulm University Medical Center, Germany; final concentration 20 μl/ml). As positive control, buffy coat cells of healthy donors were stimulated. Stimulation was performed in triplicates or at least in duplicates (dependent on available cell-counts) in 96-well flat bottom plates at 37 °C in 5% CO_2_ for 5 days. After incubation with radioactive H_3_-thymidine for 18 h (Perkin Elmer, Germany; final concentration 0.001 mCi/ml), the cells were harvested, and the incorporation in the DNA was measured in a beta counter (Microplatecounter TopCount NXT, Packard/Perkin Elmer). For each stimulated well, an index to the background count of the negative control was calculated.

From the same PBMCs, the lymphocyte subgroups were determined by flow cytometry (Navios, Beckman-Coulter) and the counts of lymphocytes (differential blood count with Sysmex; Institute for Clinical Chemistry, University of Ulm*)* and T cells (CD3+) were compared. Used antibodies for the flow cytometry are CD3-APC and CD45-Krome Orange (Beckman Coulter).

Data were collected and statistically analyzed using Excel 2013 and Word 2013 (Microsoft Office 2013). The statistical evaluation was performed via Kruskal-Wallis test and Mann-Whitney *U* test using a bilateral level of significance of *α* = 5%.

The patients were divided into three groups according to their IS medication at the time of blood taking. Group 1 was under high-level IS, meaning high doses of disease-modifying drugs (AZA, sirolimus, and tacrolimus) or any use of biologicals (adalimumab, golimumab, infliximab—which all block pro-inflammatory TNF-α—and the integrin antagonist vedolizumab) as described elsewhere [3, 9]. Group 2 was under low-level IS, which meant lower doses of disease-modifying drugs (for dosage distinction between high-level and low-level IS, see Additional file 1: Table S1). Group 3 was not treated with systemic IS therapy.

## Results

The patient characteristics and immunosuppressive therapy are demonstrated in Table 2. Most patients in group 1 were on long-term therapy with biologicals, and some even received a combination therapy with azathioprine (AZA), tacrolimus or sirolimus; all but 1 patient in group 2 are treated with AZA, and all in group 3 had no IS. All patients receiving infliximab therapy responded on induction therapy and received long-term infliximab treatment adjusted to their trough levels for dose or interval (Table 1). Three patients with low infliximab levels received combination therapy (group 1). One patient had insufficient trough levels for the first time. None of the patients receiving AZA needed dose reduction due to reduced thiopurine methyltransferase (TPMT) enzyme activity. Most patients were in clinical remission, and only 4 patients had mild disease activity at the time of measurement.

**Table 2:** Descriptive comparison of in vitro reactivity of lymphocytes

Stimulation index	PHA			Tetanus antigen			Adenovirus antigen		
	Median	Q1	Q3	Median	Q1	Q3	Median	Q1	Q3
Group 1 (*n* = 17)	306	238	955	4.2	1.6	12.8	15.3	5.0	214.3
Group 2 (*n* = 8)	442	409	483	2.7	1.7	98.1	62.5	9.9	90.5
Group 3 (*n* = 6)	962	689	1011	72.7	6.0	192.0	51.0	13.0	101.0
All samples	472	282	978	4.2	1.7	24.1	41.1	5.1	133.4

**Fig. 1:**
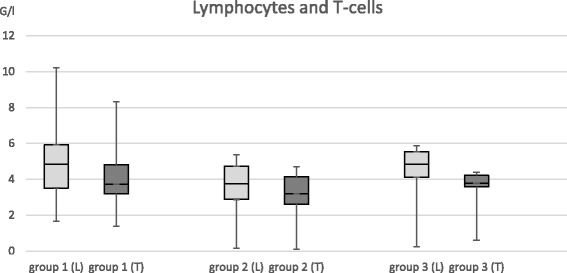
Comparison of lymphocyte and T cell counts. The vertical axis shows absolute numbers of lymphocytes and T cells in G/l. The boxplots indicate Q1, median, and Q3 with the minimum and maximum as error bars. Light gray boxplots display the lymphocytes (L) and dark gray ones the T cells (T)

The counts of lymphocytes and CD3+ T cells were measured by flow cytometry and compared between the three groups (Fig. 1). The Kruskal-Wallis test showed no significant difference between the three groups for both lymphocytes and T cells. There is not even a trend towards lower counts with higher IS (Fig. 1). Only 4 patients had low lymphocyte counts compared to age-matched references, but no patient had low T cells. The stimulation indices of these 4 patients did not differ from the rest of the collective.

The stimulation indices for the three different stimulants (PHA, tetanus antigen, adenovirus antigen) in medians, 25% quartiles (Q1), and 75% quartiles (Q3) are shown in Table 2.

**Fig. 2:**
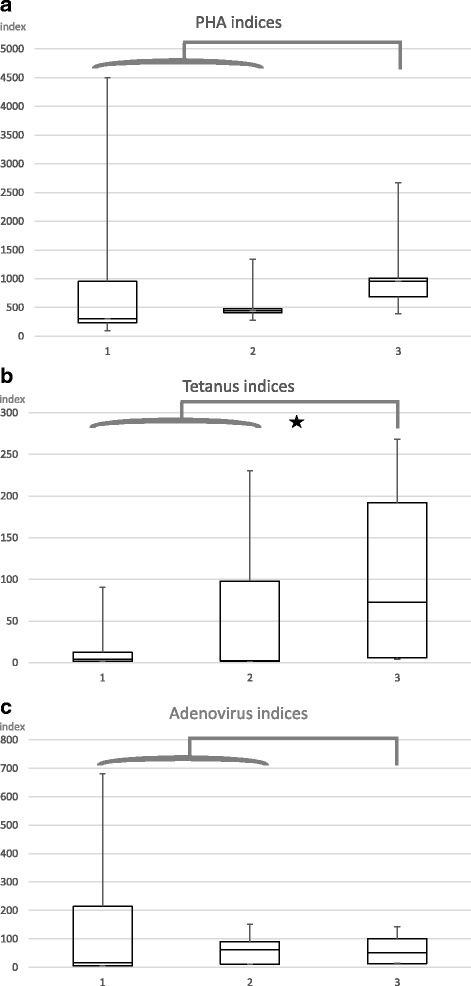
In vitro lymphocyte reactivity after stimulation with PHA, tetanus antigen, and adenovirus antigen. Lymphocytes were stimulated in vitro with PHA (**a**), tetanus antigen (**b**), and adenovirus antigen (**c**) as described. The vertical axis shows stimulation indices which are calculated as count of stimulated cells divided through count of background. The boxplots indicate Q1, median, and Q3 with the minimum and maximum as error bars. The bracket above groups 1 and 2 with the star in **b** indicates significance compared to group 3 in Mann-Whitney *U* test with bilateral level of significance of *α* = 5%, but not in **a** and **c**

We found lower stimulation indices in patients receiving IS than in those without IS. Even though a trend to higher reactivity in patients without IS can be seen, also, the groups with IS develop a notable reaction. However, the Kruskal-Wallis test could not show a significant difference in the small sample sizes between the three groups for the three different stimulants. Therefore, we compared all patients receiving IS (groups 1 + 2) with patients without IS (group 3) and performed Mann-Whitney *U* tests (Fig. 2a–c). No statistical significant difference was found for PHA and adenovirus antigen, but for tetanus antigen. The statistical test results and limits of significance are depicted in Additional file 2: Table S2.

## Discussion

Here, we show that the individual immunoreactivity in patients with IBD or AIH depending on long-term IS mono- or combo-therapy is not markedly disturbed. Indeed, one might have expected a strong difference, as immunosuppressants interact directly with T lymphocyte function.

A sufficiently functional T lymphocyte compartment in patients receiving IS is supported by three findings.

First, the blood counts of lymphocytes and T cells of the patients did not significantly differ between the three groups of high-level, low-level, and no IS.

Second, the unspecific T lymphocyte-stimulating agent PHA provoked effective proliferation in all three groups. There was no significant difference found neither in comparison of the three groups nor in comparison of all immunosuppressed patients to those without systemic IS.

Third, a specific T lymphocyte proliferation response to antigens, such as tetanus and adenovirus antigens, could be measured in patients receiving IS.

Moreover, after stimulation with adenovirus antigen, the proliferation indices showed nearly no difference at all between the three groups or in comparison of IS (groups 1 + 2) versus without IS (group 3). Indeed, group 3 is not a healthy control, but being patients in clinical remission and without any IS medication, we suggest that their immunoreactivity is very close to the one of a healthy individual.

Stimulation with tetanus antigen differs not significantly between the three groups. However, we found a trend to lower medians in patients with higher IS resulting in a significant difference in comparison of the immunosuppressed (groups 1 + 2) versus without IS (group 3).

Regarding the small number of investigated patients, we admit that we may indeed underestimate some statistical differences in these groups. However, the data are sufficient to deduct that there is a reduced but notable immunoreactivity towards different stimuli in patients with IBD or AIH under IS therapy, at least when counts of blood lymphocytes and T cells are within a normal range. In animal studies, a dependency of lymphocyte proliferation on the blood level of immunosuppressants has been demonstrated [10, 11]. Our patients were treated in effective doses to maintain remission of their disease, and the lymphocyte proliferation was still remarkably good.

In this study, most patients were in remission of their disease. In another study, patients with highly active colitis treated with infliximab in vitro were analyzed and a reduced T cell activation and proliferation was found [12]. Thus, active disease may influence T cell response in vitro.

In a study by Salinas et al., patients with rheumatic diseases receiving anti-TNF-α treatment were vaccinated against hepatitis B and pneumococcal polysaccharides. Interestingly, they observed an increase of B cell activation after TNF blockade associated with an impaired T cell-dependent humoral immune response towards hepatitis B vaccination, whereas the T cell-independent reaction to pneumococcal polysaccharides was only modestly altered [13]. It highlights that each immunosuppressant drug may provoke different immune reactions dependent on the stimulus in immunosuppressed patients.

Abnormal T cell responses to antigens are considered as a diagnostically more sensitive test of aberrant T cell function [14]. As a result of repeated childhood vaccinations, a robust T cell-specific response to tetanus antigen is expected in healthy children and adolescents. Comparing the results for antigen-specific stimulation, we found considerable differences. Especially, the adenovirus antigen stimulation provoked almost the same reaction in all groups, whereas the stimulation with tetanus antigen was found to be less effective in blood samples of immunosuppressed patients. However, other studies that compared the lymphocyte proliferation after in vitro addition of different immunosuppressants found no difference between stimulation with tetanus antigen and other stimulants [15, 16].

Although the patients in this study received a variety of disease-modifying drugs and biologicals, we found only minor differences between these groups. The results for other drugs like mycophenolate mofetil could be less promising, as a study with mycophenolate mofetil demonstrates a dose-dependent reduction in lymphocyte proliferation [17]. After treatment with AZA, which many patients in our study received, T cells seem to react well, even to suboptimal concentrations of mitogens, while cells treated with prednisolone reacted less, if the mitogen concentrations were lower [18]. It was also shown that AZA impairs lymphocyte proliferation less than cyclosporine or methylprednisolone [19]. A study with cats’ blood demonstrated equal dose-dependent inhibition of lymphocyte proliferation after in vitro addition of six different IS drugs, not including AZA [20].

Our findings of immunoreactivity in immunosuppressed patients are also supported by the fact that most live vaccinations reported in patients until yet were found to be safe and effective [21]. Still, there occurred some adverse events, which makes it important to further investigate on how and when live vaccinations under IS therapy are advisable [21]. Therefore, determining the individual immune competence of patients receiving IS before live vaccinations can be helpful in the process of individual risk-benefit analysis. In addition, to the in vitro lymphocyte proliferation assay for example, a negative QuantiFERON test with positive interferon control could also be used to prove a certain responsiveness of an individual patient’s lymphocytes [22].

## Conclusions

In this study, we examined the individual immunoreactivity of patients with IBD or AIH under IS therapy with AZA, sirolimus, tacrolimus, and different biologicals. They showed only little differences in the reaction to stimulation with different antigens. This could implicate that patients under IS therapy with these immunosuppressants can be expected to react sufficiently to at least viral antigens. Understanding the degree of specific host immune response towards a variety of immunosuppressive drugs is important and has clinical implications. Given the lack of in vivo data on the safety and efficacy of several vaccines in the growing population of immunocompromised children and adolescents, additional research is needed to guide rational recommendations.

## Additional file

Additional file 1: Table S1. Definition of high-level IS. (DOC 30 kb)
Additional file 2: Table S2. Statistical tests and results. (PDF 130 kb)
Additional file 3: Table S3. Raw data of lymphocyte proliferation assay shown as indices from duplicates/triplicates. (PDF 700 kb)

## Abbreviations

AIH: Autoimmune hepatitis
AZA: Azathioprine
CD: Crohn’s disease
CD: Cluster of differentiation
CO_2_: Carbon dioxide
CTL: Cytotoxic T lymphocyte
HFF: Human foreskin fibroblasts
IBD: Inflammatory bowel disease
IS: Immunosuppression/immunosuppressive
L: Lymphocytes
PBMC: Peripheral blood mononuclear cells
PHA: Phytohemagglutinin
Q1: 25% quartile
Q3: 75% quartile
T: T cells
TPMT: Thiopurine methyltransferase
UC: Ulcerative colitis
