# Supplementary material for: Preserved in vitro immunoreactivity in children receiving long-term immunosuppressive therapy due to inflammatory bowel disease or autoimmune hepatitis
Source: Mol Cell Pediatr. 2018 Jan 19;5:1. doi: 10.1186/s40348-018-0079-0 (PMC5775189; doi:10.1186/s40348-018-0079-0)
Supplement: Additional file 1: Table S1. — Definition of high-level IS. (DOC 30 kb) [file 40348_2018_79_MOESM1_ESM.doc]

Table S1: Definition of high-level IS

| **Medication** | **Limit for high-level IS** |
| --- | --- |
| **Azathioprine (AZA) [3]** | >3mg/kg/d |
| **Sirolimus** | blood level ≥ 8ng/ml |
| **Tacrolimus** | blood level ≥ 8ng/ml |
| **Adalimumab** | any use |
| **Golimumab** | any use |
| **Infliximab** | any use |
| **Vedolizumab** | any use |
