# Supplementary material for: Preserved in vitro immunoreactivity in children receiving long-term immunosuppressive therapy due to inflammatory bowel disease or autoimmune hepatitis
Source: Mol Cell Pediatr. 2018 Jan 19;5:1. doi: 10.1186/s40348-018-0079-0 (PMC5775189; doi:10.1186/s40348-018-0079-0)
Supplement: Additional file 2: Table S2. — Statistical tests and results. (PDF 130 kb) [file 40348_2018_79_MOESM2_ESM.pdf]

Table S2: Statistical tests and results

**Subject**	**Test**	**Groups**	**Limits for significance**	**Value of H/z**	**significant yes/no**
**lymphocyte numbers**	Kruskal-Wallis-Test	1;2;3	H>5,99	3,11	no
**T-cell numbers**	Kruskal-Wallis-Test	1;2;3	H>5,99	1,08	no
**PHA**	Kruskal-Wallis-Test	1;2;3	H>5,99	3,06	no
**PHA**	Mann-Whitney-U-Test	1+2;3	z<-1,96 or z>1,96	-1,75	no
**tetanus antigen**	Kruskal-Wallis-Test	1;2;3	H>5,99	2,89	no
**tetanus antigen**	Mann-Whitney-U-Test	1+2;3	z<-1,96 or z>1,96	-2,2	yes
**adenovirus antigen**	Kruskal-Wallis-Test	1;2;3	H>5,99	0,41	no
**adenovirus antigen**	Mann-Whitney-U-Test	1+2;3	z<-1,96 or z>1,96	-0,6	no
