# Supplementary material for: Preserved in vitro immunoreactivity in children receiving long-term immunosuppressive therapy due to inflammatory bowel disease or autoimmune hepatitis
Source: Mol Cell Pediatr. 2018 Jan 19;5:1. doi: 10.1186/s40348-018-0079-0 (PMC5775189; doi:10.1186/s40348-018-0079-0)
Supplement: Additional file 3: Table S3. — Raw data of lymphocyte proliferation assay shown as indices from duplicates/triplicates. (PDF 700 kb) [file 40348_2018_79_MOESM3_ESM.pdf]

Table S3: Data shown as indices from duplicates/triplicates

IS group	PHA index	Tetanus index	Adenovirus index
**1**	204,5	6,3	2,7
**1**	167,9	21,3	214,3
**1**	1002,0	0,7	5,0
**1**	264,0	15,6	473,3
**1**	301,1	1,8	10,0
**1**	142,0	12,8	252,2
**1**	954,8	2,3	12,0
**1**	1640,1	0,9	15,3
**3**	2674,1	132,2	61,0
**3**	613,8	212,0	141,9
**1**	505,3	90,9	1,5
**1**	4501,6	1,6	5,0
**2**	442,3	57,6	5,1
**1**	825,7	7,0	1,7
**2**	472,8	0,6	8,3
**2**	366,5	219,9	151,0
**1**	238,1	27,0	124,9
**3**	295,1	13,3	0,8
**2**	133,7	230,7	37,2
**3**	1011,3	1,6	41,1
**2**	512,4	0,8	87,8
**1**	305,7	1,7	5,2
**2**	441,3	2,0	10,5
**2**	1341,9	2,1	90,2
**3**	1010,9	268,0	114,3
**3**	912,3	3,6	3,7
**1**	268,5	7,1	266,4
**2**	423,0	3,3	91,3
**1**	234,9	1,1	117,3
**1**	1012,2	0,7	680,1
**1**	752,8	4,2	172,9
